# Magnetic resonance imaging parameters on lacrimal gland in thyroid eye disease: a systematic review and meta-analysis

**DOI:** 10.1186/s12886-023-03008-x

**Published:** 2023-08-08

**Authors:** Nicole Tsz Yan Wong, Ka Fai Kevin Yuen, Fatema Mohamed Ali Abdulla Aljufairi, Kenneth Ka Hei Lai, Zhichao Hu, Karen Kar Wun Chan, Clement Chee Yung Tham, Chi Pui Pang, Kelvin Kam Lung Chong

**Affiliations:** 1 Department of Ophthalmology and Visual Sciences, Faculty of Medicine, The Chinese University of Hong Kong, Hong Kong Special Administrative Region, Hong Kong, China; 2 Department of Ophthalmology and Visual Sciences, Prince of Wales Hospital, Hong Kong Special Administrative Region, Hong Kong, China; 3 Department of Ophthalmology, Salmaniya Medical Complex, Government Hospitals, Manama, Bahrain; 4 Department of Ophthalmology, Tung Wah Eastern Hospital, Hong Kong Special Administrative Region, Hong Kong, China; 5 Hong Kong Eye Hospital, Hong Kong Special Administrative Region, Hong Kong, China

**Keywords:** Thyroid eye disease, Thyroid-associated ophthalmopathy, Graves’ ophthalmopathy, Graves’ orbitopathy, lacrimal gland, Magnetic resonance imaging, Systematic review, Meta-analysis

## Abstract

**Background:**

Thyroid eye disease is an extrathyroidal manifestation of Graves’ disease and is associated with dry eye disease. This is the first systematic review and meta-analysis to evaluate the role of magnetic resonance imaging lacrimal gland parameters in thyroid eye disease diagnosis, activity grading, and therapeutic responses prediction.

**Methods:**

Up to 23 August, 2022, 504 studies from PubMed and Cochrane Library were analyzed. After removing duplicates and imposing selection criteria, nine eligible studies were included. Risk of bias assessment was done. Meta-analyses were performed using random-effect model if heterogeneity was significant. Otherwise, fixed-effect model was used. Main outcome measures include seven structural magnetic resonance imaging parameters (lacrimal gland herniation, maximum axial area, maximum coronal area, maximum axial length, maximum coronal length, maximum axial width, maximum coronal width), and three functional magnetic resonance imaging parameters (diffusion tensor imaging-fractional anisotropy, diffusion tensor imaging-apparent diffusion coefficient or mean diffusivity, diffusion-weighted imaging-apparent diffusion coefficient).

**Results:**

Thyroid eye disease showed larger maximum axial area, maximum coronal area, maximum axial length, maximum axial width, maximum coronal width, diffusion tensor imaging-apparent diffusion coefficient/ mean diffusivity, and lower diffusion tensor imaging-fractional anisotropy than controls. Active thyroid eye disease showed larger lacrimal gland herniation, maximum coronal area, diffusion-weighted imaging-apparent diffusion coefficient than inactive. Lacrimal gland dimensional (maximum axial area, maximum coronal area, maximum axial length, maximum axial width, maximum coronal width) and functional parameters (diffusion tensor imaging-apparent diffusion coefficient, diffusion tensor imaging-apparent diffusion coefficient) could be used for diagnosing thyroid eye disease; lacrimal gland herniation, maximum coronal area, and diffusion-weighted imaging-apparent diffusion coefficient for differentiating active from inactive thyroid eye disease; diffusion tensor imaging parameters (diffusion tensor imaging-fractional anisotropy, diffusion tensor imaging-mean diffusivity) and lacrimal gland herniation for helping grading and therapeutic responses prediction respectively.

**Conclusions:**

Magnetic resonance imaging lacrimal gland parameters can detect active thyroid eye disease and differentiate thyroid eye disease from controls. Maximum coronal area is the most effective indicator for thyroid eye disease diagnosis and activity grading. There are inconclusive results showing whether structural or functional lacrimal gland parameters have diagnostic superiority. Future studies are warranted to determine the use of magnetic resonance imaging lacrimal gland parameters in thyroid eye disease.

**Supplementary Information:**

The online version contains supplementary material available at 10.1186/s12886-023-03008-x.

## Introduction

Thyroid eye disease (TED), also known as thyroid-associated ophthalmopathy (TAO), Graves’ ophthalmopathy (GO), or Graves’ orbitopathy, is an autoimmune disorder involving the orbital soft tissues, namely the extraocular muscles (EOMs) and orbital fat (OF) [1]. It is an extrathyroidal manifestation of Graves’ disease (GD) [2]. In GD, thyrotropin receptor (TSHr) autoantibodies (TRAbs) and insulin-like growth factor-1 (IGF-1) receptor (IGF-1r) autoantibodies attack their respective receptors on orbital fibroblasts and EOMs, stimulating adipogenesis and inflammation. The resultant increased volume of retrobulbar soft tissues within the limited space contributes to various thyroid eye signs [3].

TED is a biphasic disease that begins with an active phase with progressive inflammation, followed by an inactive phase with stable fibrosis of orbital soft tissues [4]. After the current clinical assessment of TED, its differentiation of disease activity and severity is commonly based on the European Group of Graves’ Orbitopathy (EUGOGO) [5] classification system, in which the disease activity is assessed by the modified Clinical Activity Score (CAS), including spontaneous and gaze-evoked orbital pain, eyelid swelling and erythema, conjunctival erythema and chemosis, as well as inflammation of caruncle. A cutoff score of three or above out of seven items is defined as active ophthalmopathy; while less than three is inactive. TED is then classified into mild, moderate-to-severe, and sight-threatening [6]. This classification often guides TED management. However, the different clinical presentations in Asian population would raise questions whether implementing this Caucasian based classification may delay and underestimate TED diagnosis in this group, and whether there are any better potential parameters to help in early TED diagnosis.

There is currently an increasing number of studies supporting the correlation between LG dysfunction and TED progress, ranging from LG enlargement clinically [7], TSHr on LG and the involvement of TRAbs immunologically [8], LG inflammation pathologically [9], to increased proinflammatory cytokines and proteomic changes in tear films molecularly [10–13]. Recently, imaging has been acting as an adjunct to the clinical-endocrinological assessment for the diagnosis, grading, treatment, and monitoring of TED. With the established role of LG in TED, imaging studies have been changing their focus from the traditional retrobulbar soft tissues to the structural and functional changes of LG. Previously, computed tomography (CT) studies reported an increase in LG dimensions and volume in TED patients. When compared to CT, magnetic resonance imaging (MRI) has higher soft tissue resolution without radiation.

Apart from the quantitative measurements of structural parameters like dimensions [14–16] and LG herniations [17–19], functional parameters like signal intensity ratio (SIR) in T2-weighted imaging (T2WI) [16, 19], apparent diffusion coefficient (ADC) in diffusion-weighted imaging (DWI) [15, 20], and fractional anisotropy (FA) [21, 22] and ADC [21] or mean diffusivity (MD) [22] in diffusion tensor imaging (DTI) were investigated in multiple studies. To the best of our knowledge, there is no review on MRI parameters of LG in TED patients. We would like to explore whether this newer imaging modality (i.e. MRI), combined with LG parameters could better aid the clinical management of TED. Herein, this systematic review and meta-analysis review and report the outcomes and clinical implications of different MRI parameters on LG in TED patients.

## Methodology

Our systematic review and meta-analysis followed the PRISMA 2020 guidelines [23]. (PROSPERO registration number: CRD42022335591).

### Search strategy

**Fig. 1:**
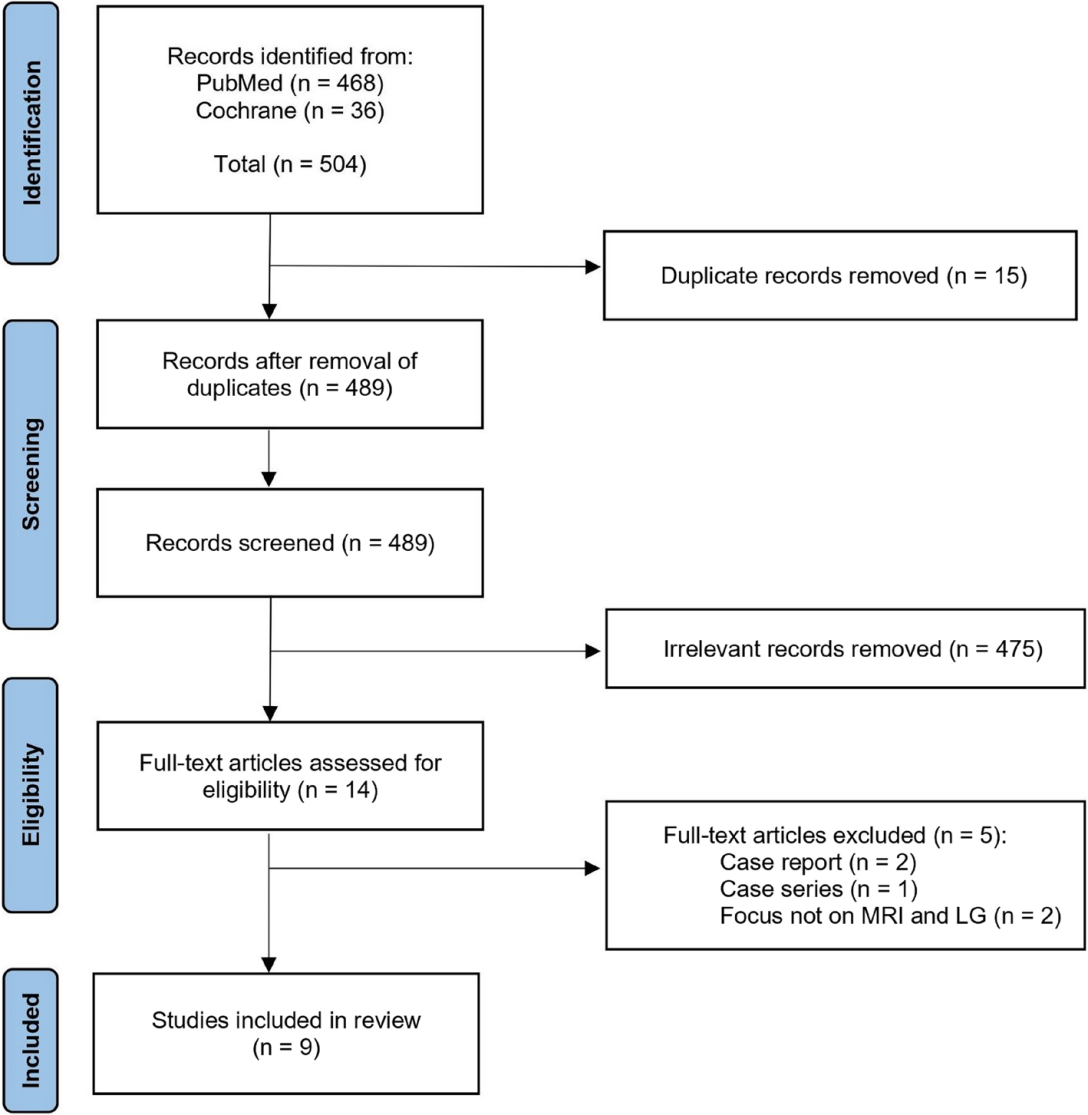
PRISMA flow diagram of literature search and selection process

On 23 August, 2022, we performed our literature search on the following electronic bibliographic databases: PubMed and the Cochrane Central Register of Controlled Trials (issue 7 of 12, July 2022). We formulated sensitive search strategies using keywords and Medical Subject Heading (MeSH) terms stated in Table 1. No language restrictions nor limitations on publication years were applied. A total of 504 results were yielded (468 from PubMed, 36 from the Cochrane Library). 15 duplicates were identified, and 489 results were left for screening (Fig. 1).

**Table 1:** Search strategies used in PubMed and Cochrane Library

Search number	Search terms
1	Graves Ophthalmopathy (MeSH Terms)
2	Graves' Ophthalmopathy (Title/Abstract)
3	Thyroid eye disease (Title/Abstract)
4	Thyroid Associated Ophthalmopathy (Title/Abstract)
5	Thyroid Associated Orbitopathy (Title/Abstract)
6	Graves’ orbitopathy (Title/Abstract)
7	Graves' eye disease (Title/Abstract)
8	#1 or #2 or #3 or #4 or #5 or #6 or #7
9	Magnetic Resonance Imaging (MeSH Terms)
10	MRI (Title/Abstract)
11	Echo-Planar Imaging (Title/Abstract)
12	Magnetic Resonance (Title/Abstract)
13	Diffusion Tensor Imaging (Title/Abstract)
14	#9 or #10 or #11 or #12 or #13
15	#8 and #14

### Selection criteria

The search mainly focused on mapping existing literature on MRI parameters on LG in TED. From the 489 results, we included studies based on the following inclusion criteria: 1) comparative studies including case–control and cohort studies, 2) cases were TED patients based on clinical diagnosis, 3) controls were healthy subjects or GD patients without TED or patients with inactive TED based on clinical diagnosis, 4) study focuses were on LG findings on MRI, 5) study subjects were unrelated individuals from clearly defined populations, 6) clear MRI LG results (or existing data adequate for calculation) in both case and control groups were provided. Animal studies, case reports, case series, reviews, abstracts, studies without or with incomplete original data were excluded.

In our first review, the titles and abstracts were screened by two independent reviewers (K.Y., and N.W.,) after applying search strategy and eligibility criteria. Disagreements were resolved after discussions with four senior reviewers (K.C., F.A., K.L., and Z.H.,). 475 irrelevant results were removed. Full text screening was then performed on the remaining 14 eligible articles by two independent reviewers (K.Y., and N.W.,). Disagreements were resolved after discussions with four senior reviewers (K.C., F.A., K.L., and Z.H.,). After ensuring eligibility, a total of nine qualified studies were included in our review and meta-analysis (Fig. 1).

### Data extraction

We adopted a pre-designed form to collect all the extracted data, including the name of first author, year of publication, country of study, ethnicity, definition of case and control groups (thyroid status), CAS, age, sex, sample size, MRI parameters and their respective findings (expressed as mean ± standard deviation (SD) or median (interquartile range (IQR)), and the purposes of the parameters used (including disease activity, severity, or therapeutic responses). We extracted and analysed data on an eye-basis instead of a patient-basis. If results were reported on a patient-basis, we estimated and converted to eye-basis based on the assumption that all subjects had two eyes. If no extractable MRI results were obtained from an eligible study, or if there were confusions with the data reported, we emailed the authors for the missing data and for verification. Two independent reviewers (K.Y., and N.W.,) extracted data, and discrepancies were resolved after discussions with four senior reviewers (K.C., F.A., K.L., and Z.H.,).

### Risk of bias (quality) assessment

We adopted a modified Newcastle–Ottawa Scale (NOS) for cross-sectional studies in the assessment of the quality of each of the selected studies [24, 25]. We gave scores based on the selection, comparability, and outcome to each study. The number of scores were marked in the same form for collecting extracted data. Any study obtaining less than or equal to six out of ten scores was considered as a high risk of inducing bias. Two independent reviewers (K.Y., and N.W.,) were involved in the quality assessment. Disagreements were resolved after discussions with four senior reviewers (K.C., F.A., K.L, and Z.H.,).

### Statistical analysis

Review Manager (RevMan, Version 5.4. The Cochrane Collaboration, 2020.) was used to perform the meta-analysis for outcome measures which were included in two or more studies. We analyzed LG herniation (LGH), LG dimensions (maximum axial area (MAA), maximum coronal area (MCA), maximum axial length (MAL), maximum coronal length (MCL), maximum axial width (MAW), maximum coronal width (MCW)), DTI-FA, DTI-ADC/MD and DWI-ADC as continuous variables. As all outcomes in the included studies were measured in the same scale, we used mean difference as the summary effect measure for all variables. Mean difference is the difference between the mean of two groups [26]. It is interpreted with *P-*value and 95% confidence interval (CI). If median and interquartile range were used in the studies, mean and standard deviation (SD) were estimated respectively as suggested by Wan et al. [27]. (See *Formula (1)* and *(2)*, Additional file 2) If the measurement of right eye and left eye were grouped and reported separately in the studies, we combined the two subgroups [26] (See *Formula (3)* *,* Additional file 2).

Heterogeneity was tested using Cochran's Q-statistics chi-square test and I^2^-statistic. If significant heterogeneity was found between the studies (*P* < 0.1 or *I*^*2*^ ≥ 50%), a random-effect model was used for meta-analysis. Otherwise, a fixed-effect model was used.

## Results

From our literature search, we identified a total of 504 titles and abstracts, and retrieved 14 full texts for review. We finally included nine studies in our systematic review and meta-analyses [14–22].

### Characteristics of included studies

Table 2 summarizes the characteristics of the nine included studies. A total of 1012 eyes were included in the nine studies, in which 693 were cases and 319 were controls. Seven studies were conducted in China recruiting Chinese subjects [14–16, 18, 19, 21], while the remaining two studies were conducted in Italy and Egypt recruiting Italians [17] and North Africans subjects [22] respectively. The age ranged from 33.5 [20] to 54.1 [18], while the sample sizes ranged from 64 [17] to 222 eyes [15]. Given that the CAS of a study [15, 17] could not be retrieved, the CAS ranged from 1 [14, 16, 17] to 4.6 [21]. Around 78% (seven out of nine) studies discussed diagnostic purposes [14–18, 20, 21], in which four studies compared both active and inactive TED with healthy controls (HCs) [14, 16, 20, 21], two studies compared active TED with inactive TED [17, 18], while one study used GD as control to make comparisons with both active and inactive TED [15]. The other two studies focused on grading [22] and therapeutic purposes [19] respectively; whilst the former compared mild and moderate-to-severe TED with HCs, the latter compared responsive to unresponsive group to glucocorticoid (GC) therapy in patients with active and moderate-to-severe TED. In terms of the MRI parameters used, three studies [14–16] looked into LG dimensions, among which all three studies reported MCA, MCL, and MCW, while only two studies [14, 16] reported MAA, MAL and MAW. Two studies investigated LG herniation in T2WI-fat suppression (T2WI-FS) [17, 18], two studies explored SIR in T2WI [16, 19], and two studies studied DWI-ADC [15, 20]. For the two studies that studied DTI-FA [21, 22], one study reported DTI-ADC [21], while the other reported DTI-MD [22].

**Table 2:** Characteristics of included studies in our systematic review and meta-analysis

**No.**	**Author (year)**	**Country**	**Ethnicity**	**Definition**		**CAS (Mean ± SD) or (Median [IQR])**		**Age (Mean ± SD) or (Median [IQR])**		**Gender (M/F)**		**Sample size**^a^** (cases/controls)**	**MRI parameters used**	**Purpose**
				**Cases**	**Controls**	**Cases**	**Controls**	**Cases**	**Controls**	**Cases**	**Controls**			
1	D. Huang (2014)	China	Chinese	TED (active + inactive)	HCs	Active: 4 (3–7) Inactive: 1 (0–2)	/	Active RE: 42.69 ± 11.593 Active LE: 42.71 ± 12.238 Inactive RE: 37.91 ± 10.406 Inactive LE: 37.40 ± 8.947	40.75 ± 11.947	Active RE: 4/9^ Active LE: 5/9^ Inactive RE: 4/7^ Inactive LE: 3/7^	8/8	48/32 (27 active + 21 inactive)/32	Dimensions (MAA, MCA, MAL, MCL, MAW, MCW)	Diagnostic
2	C. Gagliardo (2020)	Italy	Italians	TED (active)	TED (inactive)	Active: 4.2 ± 1.93 Inactive: 1 ± 0.91	/	52 [35—68]^	43 [30—64]^	5/11	5/11	32/32	T2WI-FS—LGH	Diagnostic
3	L. Chen (2021)	China	Chinese	TED (active + inactive)	HCs	Active: 4.6 ± 0.8 Inactive: 1.3 ± 0.8	/	46.4 ± 13.4 Active: 47.8 ± 9.9 Inactive: 44.9 ± 16.4	46.5 ± 13.5	12/18 Active: 7/8 Inactive: 5/10	6/9	60/30 (30 active + 30 inactive)/30	DTI – FA DTI—ADC	Diagnostic
4	D. Wu (2021)	China	Chinese	TED (active + inactive)	GD without TED	/	/	46.98 ± 12.45	36.67 ± 11.16	39/60	4/8	198/24 (142 active + 56 inactive)/24	Dimensions (MCA, MCL, MCW) DWI—ADC	Diagnostic
5	H. Hu (2016)	China	Chinese	TED (active + inactive)	HCs	Active: 4 ± 1 Inactive: 1 ± 1	/	48.4 ± 13.9	50.0 ± 12.9	12/21	8/16	66/48 (26 active + 40 inactive)/48	Dimensions (MAA, MCA, MAL, MCL, MAW, MCW) T2WI-FS—SIR	Diagnostic
6	Y. Gao (2022)	China	Chinese	TED (active)	TED (inactive)	4.21 ± 0.80	1.28 ± 0.69	54.1 ± 8.4	51.3 ± 14.0	13/16^	19/24^	29/43	T2WI-FS—LGH	Diagnostic
7	A. A. Razek (2019)	Egypt	Africans	TED (active + inactive)	HCs	Active: 4.4 ± 0.4 Inactive: 1.42 ± 0.3	/	38 ± 12.6	33.5 ± 1.3	17/27	8/12	88/40 (48 active + 40 inactive)/80	DWI—ADC	Diagnostic
8	H. Hu (2020)	China	Chinese	Acitve and moderate-severe TED responsive to GC therapy	Active and moderate-severe TED unresponsvie to GC therapy	3.9 ± 0.8	3.8 ± 0.9	47.9 ± 13.1	44.2 ± 12.5	9/20	10/8	58/36	T2WI-FS – LGH T2WI-FS—SIR	Therapeutic
9	L. Rui (2021)	China	Chinese	TED (mild + moderate-severe)	HCs	Mild: 2.00 (1.00, 2.00) Moderate-severe: 4.00 (3.00, 5.00)	/	Mild: 39.1 ± 12.3 Moderate-severe: 47.1 ± 14.5	35.4 ± 12.3	Mild: 9/13 Moderate-severe: 15/20	7/10	114/34 (44 mild + 70 moderate-severe)/34	DTI – FA DTI—MD	Grading

*ADC* Apparent diffusion coefficient, *CAS* Clinical activity score, *DTI* Diffusion tensor imaging, *DWI* Diffusion-weighted imaging, *F* female, *FA* Fractional anisotropy, *GC* Glucocorticoid, *GD* Graves' disease, *HCs* Healthy controls, *LE* left eye, *LGH* Lacrimal gland herniation, *M* Male, *MAA* Maximum axial area, *MCA* Maximum coronal area, *MAL* Maximum axial length, *MCL* Maximum coronal length, *MAW* Maximum axial width, *MCW* Maximum coronal width, *MD* Mean diffusivity, *RE* Right eye, *SD* Standard deviation, *T2WI-FS* T2-weighted imaging-fat suppression, *TED* Thyroid eye disease

^a^Sample size is expressed in an eye-basis

^Gender is expressed in an eye-basis

**Fig. 2:**
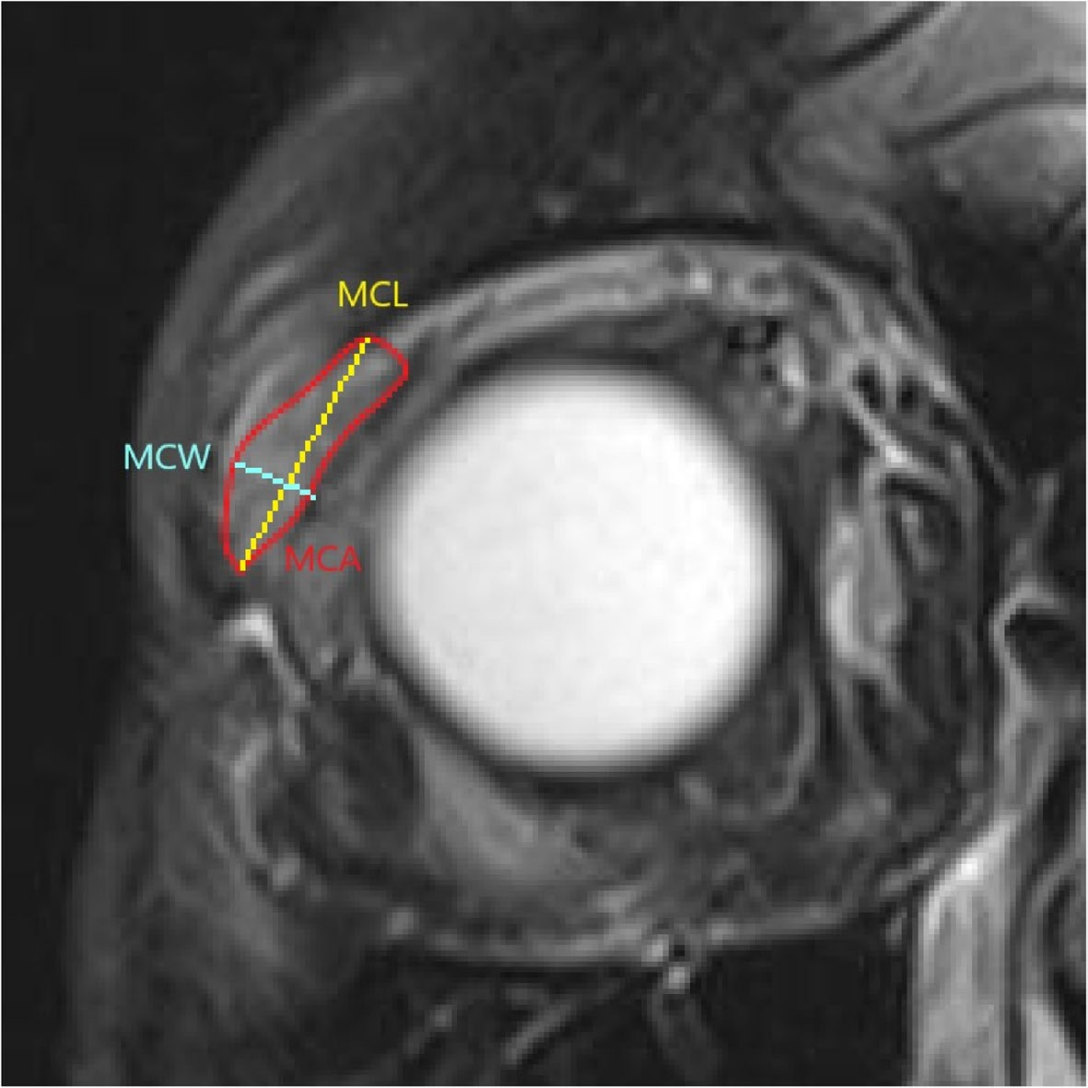
Definition of MCA, MCL and MCW. MCA = maximum coronal area; MCL = maximum coronal length; MCW = maximum coronal width

**Fig. 3:**
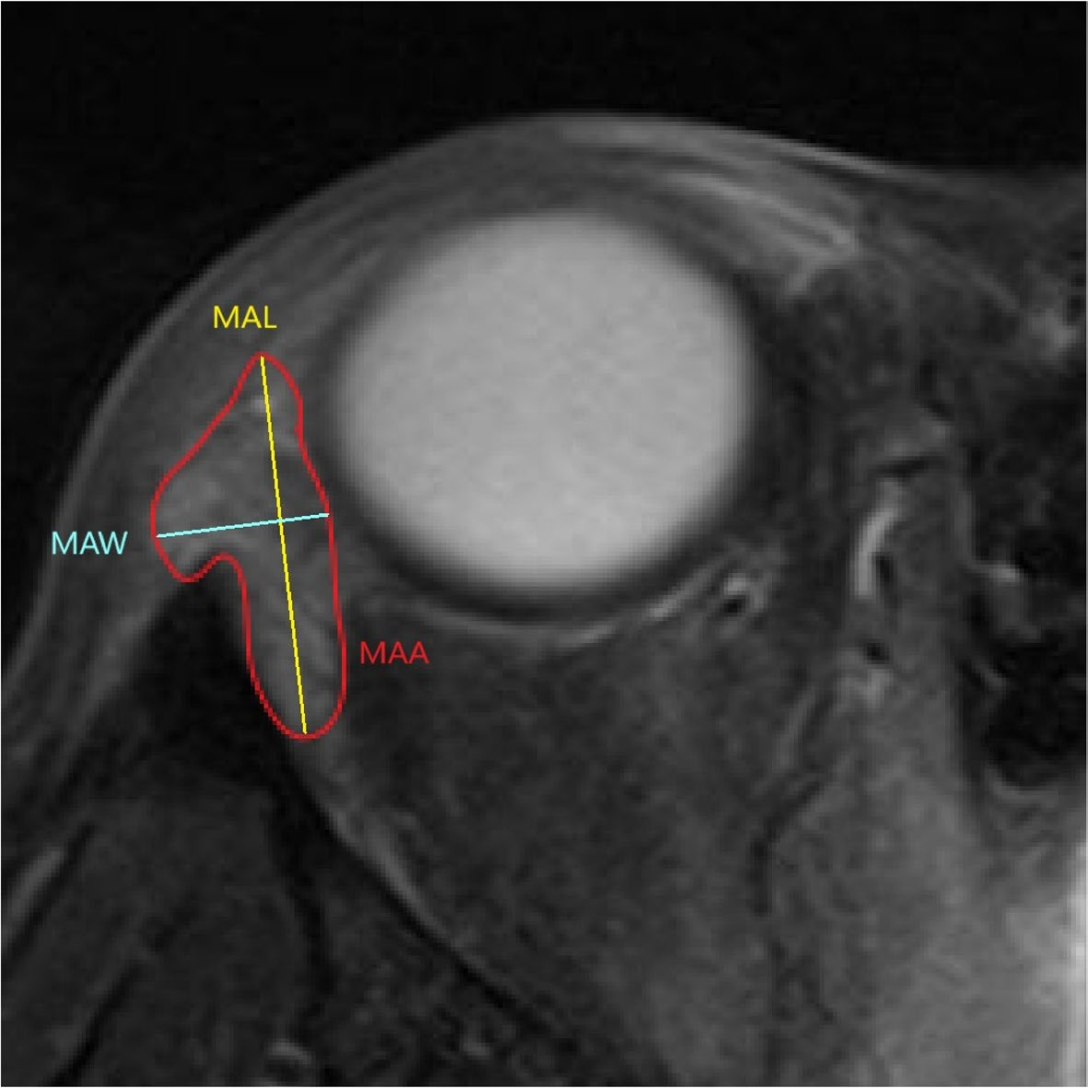
Definition of MAA, MAL and MAW. MAA = maximum axial area; MAL = maximum axial length; MAW = maximum axial width

**Fig. 4:**
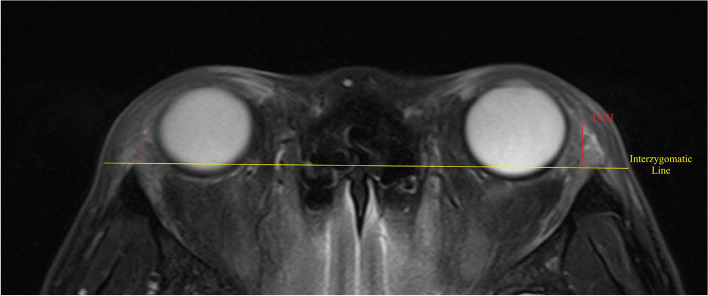
Definition of LGH. LGH = lacrimal gland herniation

The definitions of the MRI parameters are consistent among the included studies. For structural parameters, MCA is defined as LG area in the coronal image in which the LG is the largest, as shown in Fig. 2. MCL is defined as the distance between the superior tip and the inferior tip of LG in the coronal cut where MCA is obtained. MCW is defined as the widest distance perpendicular to the length (MCL) within the LG. The same principle applies to the axial parameters. MAA is defined as LG area in the axial image in which the LG is the largest, as shown in Fig. 3. MAL is defined as the distance between the anterior tip and the posterior tip of LG in the axial cut where MAA is obtained. MAW is defined as the widest distance perpendicular to the length (MAL) within the LG. LGH is defined as the distance between the anterior tip of LG and the interzygomatic line as shown in Fig. 4. For functional parameters, DWI-ADC, DTI-ADC (or MD) and DTI-FA were obtained by first placing a region of interest in the LG which has the largest cross-sectional area, and then measuring the value of ADC, MA or FA of that region of interest in DTI or DWI scan. In our paper, we combine the findings of both DTI-ADC and MD together since they both reflect the magnitude of water diffusion.

### Risks of bias in included studies

Table 3 summarized the risk of bias assessment using a modified scale adapted from the Newcastle–Ottawa Scale (NOS) for cohort studies for all our nine selected cross-sectional studies. For more in-depth details, see Supplementary Table 1, Additional File 1.

**Table 3:** Risk of bias summary

**No.**	**Author (year)**	**Selection (max 5)**				**Comparability (max 2)**	**Outcome (max 3)**		**Total no. of **
		**(1) Representativeness of the sample**	**(2) Sample size**	**(3) Non-respondents**	**(4) Ascertainment of the exposure (risk factor)**	**(1) The subjects in different outcome groups are comparable, based on the study design or analysis. Confounding factors are controlled**	**(1) Assessment of the outcome**	**(2) Statistical test**	
1	D. Huang (2014)	-	-	^1^	2	2	-	^1^	6
2	C. Gagliardo (2020)	-	-	^1^	2	2	^1^	^1^	7
3	L. Chen (2021)	-	-	^1^	2	2	2	^1^	8
4	D. Wu (2021)	-	-	^1^	2	2	2	^1^	8
5	H. Hu (2016)	1	-	^1^	2	2	**1**	^1^	8
6	Y. Gao (2022)	-	-	^1^	2	2	2	^1^	8
7	A. A. Razek (2019)	-	-	^1^	2	2	**1**	^1^	7
8	H. Hu (2020)	-	-	^1^	2	2	2	^1^	8
9	L. Rui (2021)	-	-	^1^	2	2	2	^1^	8

Details of criteria of each item could be found in supplementary materials. *Max* Maximum

Among the nine studies, except for one which scored six [14], all of them scored seven or above, indicating that they have a low risk of inducing bias [15–22]. Six of them (67%) adopted convenience sampling by choosing consecutive patients with TED [15, 17, 18, 20–22]. Two of them (22%) lacked detailed descriptions on the recruitment method of subjects [14, 22]. In terms of sample size, all studies did not justify nor show relevant sample size calculation. They all lacked an explanation on the expected sample size to provide a statistically significant information [14–22]. For outcome assessment, five of them (56%) involved more than one assessor who were blinded to the clinical condition of subjects to independently evaluate the MRI results. The intra- or inter-observer variability were appropriately adjusted using relevant statistical methods [15, 18, 19, 21, 22]. Two of them did not mention whether the assessors were blinded or not [16, 17]; while one of them involved only one blinded assessor and did not mention the correction of intra-observer variability [20]. Otherwise, all studies had satisfactory response rates and established characteristics of the subjects, included CAS to ascertain the exposure of subjects (*i.e.* the status of active or inactive TED), adjusted age and sex as confounders, and clearly stated the appropriate statistical test for data analysis [14–22].

### Outcome measures

i. **Active vs inactive**

Table 4 summarized the MRI parameters used in the included studies to compare between active TED group and inactive TED group.

**Table 4:** Summary of MRI parameters of Active TED group vs. Inactive TED group in included studies

Parameter	No.	Author (year)	Sample size (eye)			Outcome (Mean ± SD OR Median (IQR))	
			**Active**	**Inactive**	**Control**	**Active**	**Inactive**
LG herniation (mm)	#2	Gagliardo 2020	32	32	/	RE: 10.1 (7.3–17) LE: 8.5 (6.6–13)	RE: 7 (0–13.4) LE: 5.8 (0–12)
	#6	Gao 2022	29	43	/	15.5 ± 3.25	12.21 ± 2.09
T2WI-FS—SIR	#5	Hu 2016	26	40	48	2.72 ± 0.51	2.34 ± 0.36
Dimension							
Maximum axial area (mm^2^)	#1	Huang 2014	27	21	32	94.29 ± 17.29	79.92 ± 14.86
	#5	Hu 2016	26	40	48	68.9 ± 31.49	68.63 ± 20.97
Maximum coronal area (mm^2^)	#1	Huang 2014	27	21	32	103.93 ± 20.44	89.32 ± 17.36
	#4	Wu 2021	142	56	24	75 (64, 92)	66 (57, 86)
	#5	Hu 2016	26	40	48	70.36 ± 29.74	68.42 ± 17.6
Maximum axial length (mm)	#1	Huang 2014	27	21	32	18.64 ± 3.09	18.28 ± 2.35
	#5	Hu 2016	26	40	48	16.7 ± 3.7	15.9 ± 3.4
Maximum coronal length (mm)	#1	Huang 2014	27	21	32	21.67 ± 4.04	20.96 ± 3.35
	#4	Wu 2021	142	56	24	18.8 (17.4, 20.4)	18.9 (16.1, 21.0)
	#5	Hu 2016	26	40	48	16.0 ± 4.5	16.3 ± 2.8
Maximum axial width (mm)	#1	Huang 2014	27	21	32	7.21 ± 1.39	6.44 ± 1.41
	#5	Hu 2016	26	40	48	5.1 ± 1.1	6.1 ± 3.8
Maximum coronal width (mm)	#1	Huang 2014	27	21	32	6.77 ± 1.48	6.61 ± 1.3
	#4	Wu 2021	142	56	24	5.8 (4.9, 6.5)	5.6 (4.7, 5.9)
	#5	Hu 2016	26	40	48	5.4 ± 1.2	5.4 ± 0.9
DTI-FA	#3	Chen 2021	30	30	30	0.277 ± 0.04	0.349 ± 0.071
DTI-ADC (10^–3^ mm^2^/s)						1.477 ± 0.342	1.329 ± 0.164
DWI-ADC (10^–3^ mm^2^/s)	#4	Wu 2021	142	56	24	1007.08 (939.71, 1074.83)	955.89 (863.26, 1043.70)
	#7	Razek 2019	48	40	40	1.83 ± 0.08	1.69 ± 0.04

**Fig. 5:**
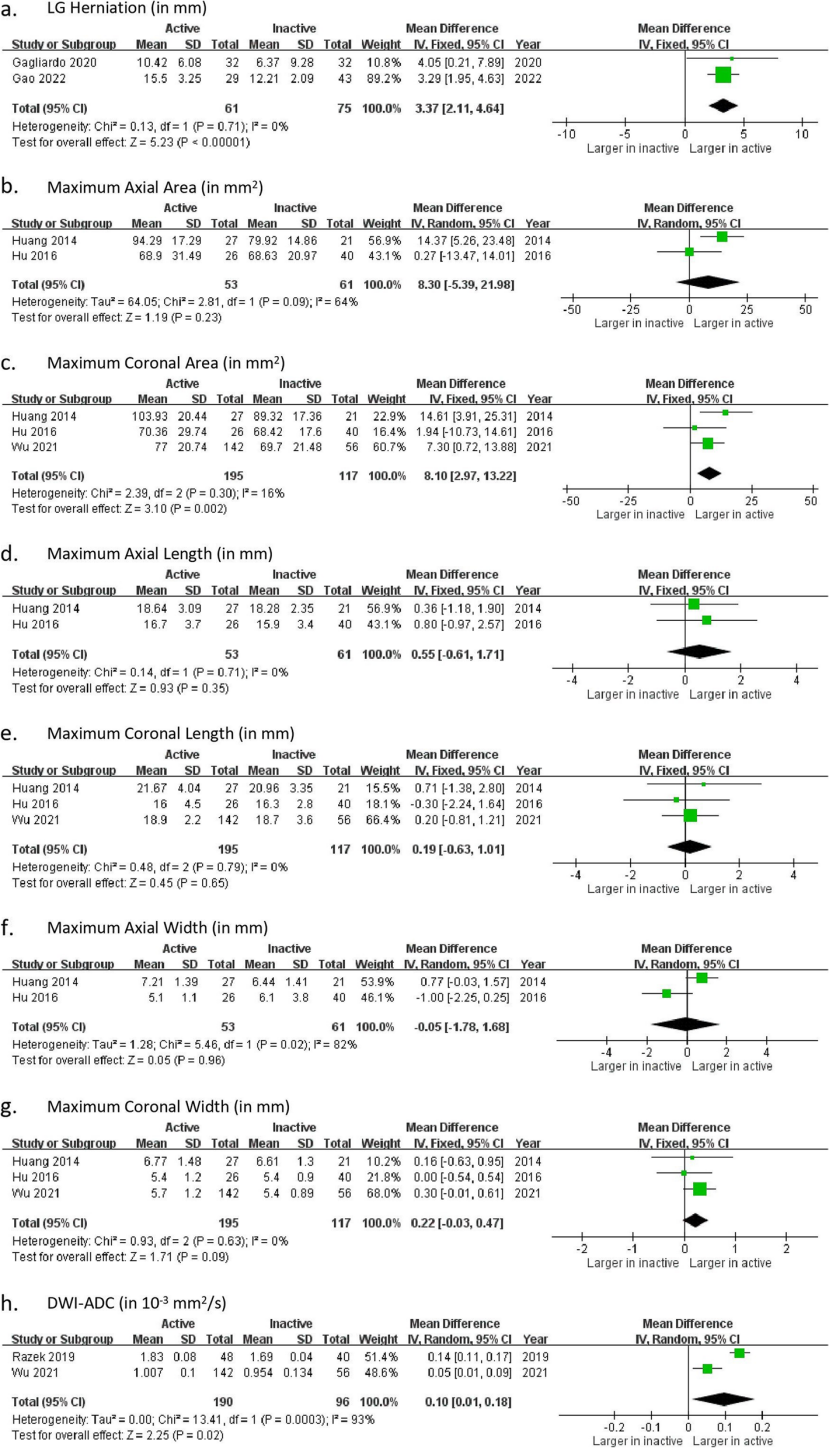
Lacrimal gland parameters in active TED and inactive TED groups. SD = standard deviation; IV = inverse variance; CI = confidence interval

We conducted a meta-analysis on eight MRI measurements of active TED patients with inactive TED patients as control group, including LG herniation, LG dimensional parameters (MAA, MCA, MAL, MCL, MAW, MCW) and DWI-ADC. The results are shown in Fig. 5, and the summary is shown in Table 5. Two to three studies were included in each outcome measures. In MAA, MAW and DWI-ADC, there were statistically significant heterogeneity.

**Table 5:** Meta-analyses of MRI parameters of Active TED group vs. Inactive TED group

Parameter	Pooled mean difference	95% CI	*P*-value	Significant difference?	I^2^
LG herniation	3.37 mm	[2.11, 4.64]	< 0.00001	Yes	0%
Dimension					
Maximum axial area	8.3 mm^2^	[-5.39, 21.98]	0.23	No	64%
Maximum coronal area	8.1 mm^2^	[2.97, 13.22]	0.002	Yes	16%
Maximum axial length	0.55 mm	[-0.61, 1.71]	0.35	No	0%
Maximum coronal length	0.19 mm	[-0.63, 1.01]	0.65	No	0%
Maximum axial width	-0.05 mm	[-1.78, 1.68]	0.96	No	82%
Maximum coronal width	0.22 mm	[-0.03, 0.47]	0.09	No	0%
DWI-ADC	0.1 × 10^–3^ mm^2^/s	[0.01, 0.18]	0.02	Yes	93%

*CI* Confidence interval

The active TED group showed a significant larger LG herniation than the inactive TED group by 3.37 mm (Fig. 5a). For LG dimensions, there was significant difference between the two groups only in MCA by 8.1 mm^2^ (Fig. 5c). In contrast, there were no significant differences in MAA (pooled mean difference: 8.3 mm^2^; Fig. 5b), MAL (pooled mean difference: 0.55 mm; Fig. 5d), MCL (pooled mean difference: 0.19 mm; Fig. 5e), MAW (pooled mean difference: -0.05 mm; Fig. 5f) and MCW (pooled mean difference: 0.22 mm; Fig. 5g). The active TED group was also associated with higher DWI-ADC than the inactive TED group by 0.1 × 10^–3^ mm^2^/s (Fig. 5h).

ii. **TED vs control**

Table 6 summarized the MRI parameters used in the included studies to compare between TED group and healthy control group.

**Table 6:** Summary of MRI parameters of TED group vs. Healthy control group in included studies

Parameter	No.	Author (Year)	Sample size (eye)			Outcome (Mean ± SD OR Median (IQR))	
			**Active**	**Inactive**	**Control**	**TED**	**Control**
T2WI-FS—SIR	#5	Hu 2016	26	40	48	2.49 ± 0.46	2.17 ± 0.32
Dimension							
Maximum axial area (mm^2^)	#1	Huang 2014	27	21	32	88 ± 17.64	65.8 ± 11.56
	#5	Hu 2016	26	40	48	68.74 ± 25.40	43.73 ± 18.57
Maximum coronal area (mm^2^)	#1	Huang 2014	27	21	32	97.54 ± 20.33	73.49 ± 12.61
	#4	Wu 2021	142	56	24	74 (60, 87)	67 (58, 81)
	#5	Hu 2016	26	40	48	69.18 ± 22.97	54.96 ± 19.11
Maximum axial length (mm)	#1	Huang 2014	27	21	32	18.48 ± 2.77	16.97 ± 2.12
	#5	Hu 2016	26	40	48	16.2 ± 3.5	14.0 ± 1.9
Maximum coronal length (mm)	#1	Huang 2014	27	21	32	21.36 ± 3.74	20.06 ± 3.05
	#4	Wu 2021	142	56	24	18.8 (17.2, 20.7)	19.7 (18.8, 20.5)
	#5	Hu 2016	26	40	48	16.2 ± 3.5	15.3 ± 2.7
Maximum axial width (mm)	#1	Huang 2014	27	21	32	6.87 ± 1.44	5.65 ± 1.12
	#5	Hu 2016	26	40	48	5.7 ± 3.1	3.8 ± 0.8
Maximum coronal width (mm)	#1	Huang 2014	27	21	32	6.7 ± 1.39	5.39 ± 1.01
	#4	Wu 2021	142	56	24	5.7 (4.9, 6.3)	4.9 (4.4, 5.8)
	#5	Hu 2016	26	40	48	5.4 ± 1.0	4.3 ± 0.7
DTI-FA	#3	Chen 2021	30	30	30	0.313 ± 0.067	0.375 ± 0.082
	#9	Rui 2021	56	58	34	0.376 ± 0.031	0.399 ± 0.049
DTI-ADC (10^–3^ mm^2^/s)	#3	Chen 2021	30	30	30	1.403 ± 0.276	1.289 ± 0.172
DTI-MD (10^–3^ mm^2^/s)	#9	Rui 2021	56	58	34	1.39 ± 0.08	1.34 ± 0.04
DWI-ADC (10^–3^ mm^2^/s)	#4	Wu 2021	142	56	24	989.81 (915.00, 1063.27)	965.56 (881.40, 1014.81)
	#7	Razek 2019	48	40	40	1.73 ± 0.08	1.52 ± 0.04

**Fig. 6:**
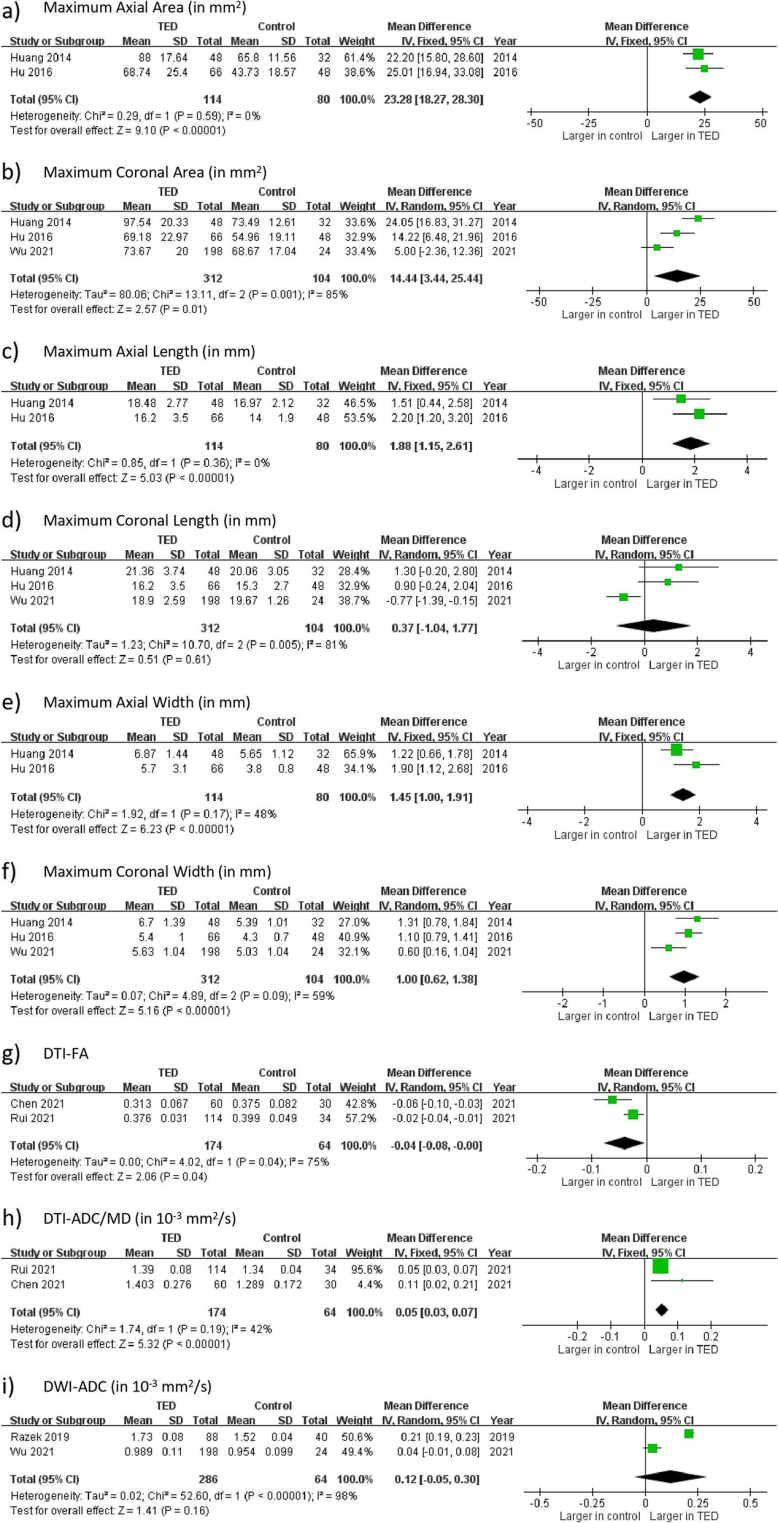
Lacrimal gland parameters in TED and control groups. SD = standard deviation; IV = inverse variance; CI = confidence interval

We also conducted a meta-analysis on nine MRI measurements of TED patients compared to control group, including LG dimensional parameters (MAA, MCA, MAL, MCL, MAW, MCW), DTI-FA, DTI-ADC/MD, and DWI-ADC. All included studies used healthy subjects as control group except Wu, who used Grave’s disease patients without TED as control [15]. The results are shown in Fig. 6, and the summary is shown in Table 7. Two to three studies were included in each outcome measures. In MCA, MCL, MCW, DTI-FA and DWI-ADC, there were statistically significant heterogeneity.

**Table 7:** Meta-analyses of MRI parameters of TED group vs. Healthy control group

Parameter	Pooled mean difference	95% CI	*P*-value	Significant difference?	I^2^
Dimension					
Maximum axial area	23.28 mm^2^	[18.27, 28.30]	< 0.00001	Yes	0%
Maximum coronal area	14.44 mm^2^	[3.44, 25.44]	0.01	Yes	85%
Maximum axial length	1.88 mm	[1.15, 2.61]	< 0.00001	Yes	0%
Maximum coronal length	0.37 mm	[-1.04, 1.77]	0.61	No	81%
Maximum axial width	1.45 mm	[1.00, 1.91]	< 0.00001	Yes	48%
Maximum coronal width	1.00 mm	[0.62, 1.38]	< 0.00001	Yes	59%
DTI-FA	-0.04	[0.00, 0.08]	0.04	Yes	75%
DTI-ADC/MD	0.05 × 10^–3^ mm^2^/s	[0.03, 0.07]	< 0.00001	Yes	42%
DWI-ADC	0.12 × 10^–3^ mm^2^/s	[-0.05, 0.30]	0.16	No	98%

For LG dimensions, there was significant difference between the TED group and the control group in MAA (pooled mean difference: 23.28 mm^2^; Fig. 6a), MCA (pooled mean difference: 14.44 mm^2^; Fig. 6b), MAL (pooled mean difference: 1.88 mm; Fig. 6c), MAW (pooled mean difference: 1.45 mm; Fig. 6e), and MCW (pooled mean difference: 1.00 mm; Fig. 6f). There was no significant difference in MCL (pooled mean difference: 0.37 mm; Fig. 6d). The TED group was associated with lower DTI-FA than the control group by 0.04 (Fig. 6g), and higher DTI-ADC/MD by 0.05 × 10^–3^ mm^2^/s (Fig. 6h). No significant difference in DWI-ADC was found (pooled mean difference: 0.12 × 10^–3^ mm^2^/s; Fig. 6i).

iii. **Other MRI parameters**

Table 8 summarized other MRI parameters used in the included studies for grading or therapeutic purposes.

**Table 8:** Summary of other MRI parameters for grading or therapeutic purposes

Sequence—Parameter	No.	Author (year)	Sample size (eye)		Purpose (diagnostic/ grading/ therapeutic)	Outcome (Mean ± SD)	
			**Group 1**	**Group 2**		**Group 1**	**Group 2**
T2WI-FS—LG herniation (mm)	#8	Hu 2020	Responsive: 58	Unresponsive: 36	Therapeutic: responsive vs unresponsive to IV steroid after 6 months	11.12 ± 2.15	9.89 ± 2.54
T2WI-FS—SIR	#8	Hu 2020	Responsive: 58	Unresponsive: 36	Therapeutic: responsive vs unresponsive to IV steroid after 6 months	SIR-max: 3.35 ± 0.88	3.44 ± 0.94
						SIR-mean: 2.42 ± 0.55	2.42 ± 0.76
						SIR-min: 1.43 ± 0.44	1.48 ± 0.53
DTI-FA	#9	Rui 2021	Moderate to Severe: 35	Mild: 22	Grading (EUGOGO): moderate to severe vs mild	0.370 ± 0.03	0.386 ± 0.031
DTI-MD (10^-3 mm^2/s)						1.41 ± 0.08	1.37 ± 0.08

## Discussion

This systematic review and meta-analysis focused on the MRI measurement on LG of TED patients. Two to three studies were included in the meta-analyses. Active TED patient group has significantly larger LGH, larger MCA and larger DWI-ADC value than inactive TED patients. TED patient group was significantly larger in five dimensional parameters (MAA, MCA, MAL, MAW, MCW) and DTI-ADC/MD, and was significantly lower in DTI-FA than health controls.

### MRI LG parameters comparisons

i. **Active TED** ***vs.*** **inactive TED**

In the comparison between active TED patients and inactive TED patients, we found that active TED patient group has significantly larger LGH, larger MCA and larger DWI-ADC value. This implies that these three parameters are potential parameters to differentiate active TED patients from the inactive ones. Out of the seven structural parameters, only LGH and MCA have significant difference. In contrast to functional MRI parameters, structural parameters measure physical LG characteristics to indirectly reflect the degree of inflammation, which is generally more severe in active TED. As a result, structural MRI parameters may not be superior to differentiate active TED patients from inactive ones, comparing with functional MRI parameters. Out of the three parameters showing significant differences, LGH (*I*^*2*^ = 0%; Fig. 5a) and MCA (*I*^*2*^ = 16%; Fig. 5c) have insignificant heterogeneity, while DWI-ADC (*I*^*2*^ = 93%; Fig. 5h) has substantial heterogeneity. The high heterogeneity may affect the validity of the result.

ii. **TED** ***vs.*** **control**

In the comparison between TED patients and control, TED patient group was significantly larger in five dimensional parameters (MAA, MCA, MAL, MAW, MCW) and DTI-ADC/MD, and was significantly lower in DTI-FA. This implies that these seven parameters are potential parameters to differentiate TED patients from healthy subjects. Five out of six structural parameters (MAA, MCA, MAL, MAW, MCW) show significant differences. As the difference in the severity of inflammation between TED patients and control is larger than that between active TED patients and the inactive ones, structural parameters can also differentiate TED patients from healthy subjects.

For dimensional parameters, all coronal parameters, *i.e.*, MCA (*I*^*2*^ = 85%; Fig. 6b), MCL (*I*^*2*^ = 81%; Fig. 6d) and MCW (*I*^*2*^ = 59%; Fig. 6f), show significant heterogeneity. In contrast, all axial parameters, *i.e.*, MAA (*I*^*2*^ = 0%; Fig. 6a), MAL (*I*^*2*^ = 0%; Fig. 6c) and MAW (*I*^*2*^ = 48%; Fig. 6e), show insignificant heterogeneity. The study by Wu only measured coronal parameters [15]. It is observed that the result of Wu’s study showed lower mean differences consistently, accounting to the high heterogeneity in coronal parameters.

iii. **Structural parameters**

Among the dimensional parameters, area parameters perform better at differentiating active TED patients from the inactive ones and TED patients from healthy subjects than length and width parameters. Only MCA can differentiate active TED patients from the inactive ones (Fig. 5c), while both MCA and MAA can differentiate TED patients from healthy subjects (Fig. 6a and 6b). Length and width parameters cannot differentiate active TED patients from the inactive ones. The possible reason is that areas are two-dimensional entities. The differences between the groups are more prominent. Area parameters are also more accurate as they reflect changes in two dimensions. LG volume may be an even better dimensional parameter because it is three-dimensional. Among the nine included studies, only the study by Hu (2016) measured LG volume [16]. Hu’s method for measuring LG volume requires delineating LG in all slides to obtain the areas, and then multiplying the sum of area and slice interval to compute the volume [16]. It is much more labour-intensive to measure volume than area, as it is required to delineate LG in all slides. In clinical practice, it is more difficult to manually measure LG volume for all patients. Measuring maximum area is easier and more practical.

Among the six dimensional parameters, the best parameter is MCA and the worst parameter is MCL. MCA can differentiate active TED patients from the inactive ones (Fig. 5c), and TED patients from healthy subjects (Fig. 6b). In contrast, MCL cannot differentiate both (Figs. 5e and 6d).

iv. **Functional parameters**

Substantial heterogeneity was observed in DWI-ADC of both comparisons (*I*^*2*^ = 93% in active TED vs. inactive TED, Fig. 5h; *I*^*2*^ = 98% in TED vs control, Fig. 6i). It is observed that DWI-ADC value in Wu’s study is generally lower than that in Razek’s study [15, 20]. Wu’s method of measuring DWI-ADC value involved delineating the largest coronal area in T2 weighted sections, and then measuring the ADC value of that area in DWI sequence. The most hyperintense spot, which represents the area of most severe inflammation, may not be hit. In contrast, Razek’s method involved placing region of interest directly in DWI sequence and measuring the ADC value. The difference in the method of measuring DWI-ADC value is a possible reason for the generally low DWI-ADC values in Wu’s study, and thus the high heterogeneity in the meta-analyses. Another possible reason is ethnicity difference. Wu’s study recruited Chinese subjects while Razek’s study recruited Egyptians [15, 20].

xxii. **Structural** ***vs.*** **functional parameters**

In evaluating the inflammatory activity, functional MRI parameters may be better than structural MRI parameters as functional parameters reflect directly on the level of metabolic activity. However, in both comparisons (*i.e.*, active TED vs inactive TED and TED vs healthy control), structural and functional MRI parameters show comparable results in differentiating between two groups. Two (*i.e.*, LGH and MCA) out of seven structural parameters, and one (*i.e.*, DWI-ADC) out of one functional parameter can differentiate active TED patients from the inactive ones. Five (*i.e.*, MAA, MCA, MAL, MAW and MCW) out of six structural parameters, and two (*i.e.*, DTI-FA and DTI-ADC/MD) out of three functional parameters can differentiate TED patients from healthy subjects. The result of this meta-analyses showed that functional MRI parameters has no superiority than structural MRI parameters, and vice versa. Computed tomography (CT) is another imaging modality that can measure structural parameters. With comparable results between functional and structural MRI parameters, CT may be comparable to functional MRI in diagnosing TED. However, a study by Lee showed the sensitivity of CT and MRI for detecting active inflammation in TED is 50% and 100% respectively, despite limited validation of the ability of MRI in the study [28]. As a result, there was an inconclusive result in evaluating the superiority between structural and functional MRI parameters.

#### Clinical implications

i. **Clinical diagnosis of disease activity**

Out of the nine included studies, seven studies (78%) were conducted on Chinese patients (Table 2). This could possibly be accounted by the more difficult diagnosis and management based on the clinical manifestations of Chinese patients with TED, where Lim et al*.* concluded that East Asians generally had fewer exophthalmos, upper eyelid retractions and edema than Caucasian patients, leading to more research interests in finding alternatives (*e.g.* imaging modalities) for earlier detection or diagnosis of TED [29]. While five [14–16, 18, 21] out of these seven studies, plus two studies done in Italy [17] and Egypt [20], looked into the different LG parameters to aid the diagnosis of TED, we are the first study to do a meta-analysis on the data provided in all these studies. We have found that the LG parameters, both structural and functional, generally provide a more significant diagnostic value in differentiating TED from disease-free patients than in differentiating active from inactive TED patients. To be more precise, LG dimensional parameters including MAA, MCA, MAL, MAW, MCW (Fig. 6a-c, e, f), as well as LG functional parameters including DTI-FA and DTA-ADC/MD (Fig. 6g, h) could possibly be used in clinical practices for differentiating TED from disease-free patients. This is compared to the fewer parameters, *i.e.*, LG herniation, MCA, and DWI-ADC (Fig. 5a, c, h), that could possibly be used to differentiate active from inactive TED patients. This implies that we might take these LG MRI parameters into account when diagnosing TED in the future along with the traditional modified CAS. The possibility of creating a new scoring system for TED activity diagnosis incorporating LG MRI parameters may also be considered, especially among the Asian population.

ii. **Grading of disease severity**

Among all the nine included studies, only a Chinese study by Rui et al*.* [22] took a step further to compare mild to moderate-severe TED patients and investigate the use of DTI parameters for grading TED severity. Thus, we could not perform a meta-analysis regarding this perspective. Based on the findings by Rui et al*.* [22], moderate-severe TED group had significantly lower DTI-FA, especially of medial rectus (MR) (*P* = 0.017), and higher DTI-MD (*P* = 0.021) than mild TED group. It also concluded that DTI parameters, especially FA, of MR were sensitive indicators that could help in the differentiation between mild and moderate-severe TED. From this result, we could see the potential role of LG DTI parameters in guiding the grading of TED severity and hence the management plan of TED patients more accurately. However, it is obvious that more studies need to be carried out to draw a more statistically significant conclusion.

iii. **Prediction of therapeutic responses and prognosis**

Similar to the above, among all the nine included studies, only a Chinese study by Hu et al*.* [19] compared the LG parameters, *i.e.*, LG herniation and SIR (SIR-max, SIR-mean, SIR-min), of active and moderate-severe TED patients responsive to intravenous (IV) steroidal therapy after six months to those unresponsive patients. Thus, a meta-analysis regarding the therapeutic responses was not performed. Based on the sole results by Hu et al*.* [19], it is found that those responsive to IV steroids had a significantly larger LG herniation than those unresponsive (*P* = 0.019), while there were no statistically significant differences in SIR (SIR-max, SIR-mean, SIR-min) between the two groups (*P* = 0.514, 0.776 and 0.642 respectively). It summarized that the larger LG herniation could possibly be used to distinguish treatment responsive and unresponsive group. This could then possibly allow a wiser allocation of treatment plans, *i.e.*, glucocorticoid therapy for responsive patients, and immunotherapies for unresponsive patients. With more studies investigating this aspect, a more accurate conclusion could then be drawn, and hence more targeted treatment plans could be made for patients to improve their disease prognosis.

### Use of (LG) imaging in managing other orbital/ inflammatory diseases

Apart from the LG parameters studied in our systematic review and meta-analysis for the diagnosis, grading, and prediction of therapeutic responses in TED, in fact, different LG parameters have gained an emerging role in the assessment of other orbital or inflammatory diseases. For instance, one of the differential diagnoses of dry eyes is primary Sjogren’s syndrome (pSS), which is an autoimmune disease affecting the salivary and LGs, causing dryness of mouth and eyes [30]. However, its clinical diagnosis is difficult due to its non-specific signs and symptoms [31]. While the current diagnostic criteria related to the orbit involves Schirmer’s test and ocular dry scores [32, 33], discomfort to patients and uneasy interpretation arises respectively [31]. Hence, different studies have tried to look for different LG parameters by non-invasive imaging to aid pSS diagnosis. For example, the change in LG size and enhanced signal intensity with accelerated fat deposition in MRI could predict pSS stages [34]; the significantly lower DWI-ADC of LG may suggest LG abnormalities in pSS patients [35]; the lower 11C-MET uptake by LG in PET-CT scan has found to have positive correlation with reduced tear flow [36]. Another example of LG-related orbital disease is IgG4-related disease (IgG4-RD), which is a fibroinflammatory disease with lymphoplasmacytic IgG4-positve plasma cells infiltration to multiple organ tissues that could involve the orbit, in case of IgG4-related ophthalmic disease (IgG4-ROD) [37]. Its current diagnosis is based on the typical organ dysfunctions or structural changes (*i.e.*, swelling), high serum IgG4 titer, and histopathological results from biopsy which is invasive, while imaging could also serve as a non-invasive tool to aid the diagnosis [38]. For instance, it is found that the hypointense and enlarged LG on T2W MRI [39], and the higher uptake of 68 Ga-FAPI in PET-CT by LG could aid the diagnosis and assessment of IgG4-RD [40]. While both pSS and IgG4-RD could result in enlarged LG, the infraorbital nerve enlargement (IONE) in MRI could act as a specific MRI sign of IgG4-ROD [41]. Besides, IONE could also help differentiate IgG4-ROD from other lymphoproliferative orbital diseases, including lymphoma, reactive lymphoid hyperplasia, and idiopathic or other orbital inflammation [42]. From these examples, we could see an increasingly important role of non-invasive imaging techniques, as well as the rising role of LG parameters on different imaging to aid the diagnosis of various orbital or inflammatory diseases in which ophthalmologists are of particular interests.

### Limitations

There were few limitations in our systematic review. First, only two to three studies were included under each outcome measures. The pooled sample sizes may not be large enough to draw a clinically significant conclusion due to random sampling error. Secondly, if heterogeneity was found to be significant by Cochran's Q-statistics chi-square test and I^2^-statistic, subgroup analysis cannot be performed as each subgroup would consist of one to two studies only. As a result, heterogeneity may be significant and may affect the validity of the result of meta-analysis. Further investigations may be needed to explore the reasons behind the high heterogeneity, such as ethnicity differences and scanner dependent differences. Thirdly, the few numbers of included studies also reflects that the field of MRI on lacrimal gland in thyroid eye disease requires further studies.

Out of the nine included studies, seven studies were written by Chinese authors and recruited Chinese subjects [14–16, 18, 19, 21, 22]. Care in application of results to other ethnicities should be considered. Previous studies have demonstrated that differences in clinical manifestations of TED exist between East Asian and Caucasian patients [29, 43]. Radiological differences can arise between different ethnicities, affecting the representativeness of this meta-analysis as most included patients are Chinese.

### Future insights

This is the first systematic review and meta-analysis on the use of MRI LG parameters in TED patients. MRI is a non-invasive imaging modality that can effectively guide the management of TED patients. While the current number of studies on MRI LG parameters is limited, where only two to three studies focus on each parameter, more studies with larger sample sizes and from a wider range of ethnicities would be warranted. The potential LG imaging markers for TED, especially in the aspects of disease grading and therapeutic responses prediction are still under investigations. The use of MRI, which is non-invasive, safe, highly sensitive, could possibly be a rising trend for the diagnosis of TED or other orbital diseases.

## Conclusions

The systematic review and meta-analyses suggest that lacrimal gland herniation, maximum coronal area, and DWI-ADC are able to detect TED patients with active diseases. Maximum axial area, maximum coronal area, maximum axial length, maximum axial width, maximum coronal width, DTI-FA, and DTI-ADC/MD are able to differentiate TED patients from healthy controls. Further studies on the use of MRI on lacrimal gland in the field of thyroid eye disease are warranted to confirm our results.

### Supplementary Information

**Additional file 1.**

**Additional file 2.**

## Data Availability

All data generated or analyzed during this study are included in this published article and its supplementary information files.

## Abbreviations

TED: Thyroid eye disease
GD: Graves’ disease
DED: Dry eye disease
MRI: Magnetic resonance imaging
LG: Lacrimal gland
LGH: Lacrimal gland herniation
MAA: Maximum axial area
MCA: Maximum coronal area
MAL: Maximum axial length
MCL: Maximum coronal length
MAW: Maximum axial width
MCW: Maximum coronal width
SIR: Signal intensity ratio
T2WI: T2-weighted imaging
ADC: Apparent diffusion coefficient
DWI: Diffusion-weighted imaging
FA: Fractional anisotropy
MD: Mean diffusivity
DTI: Diffusion tensor imaging
TAO: Thyroid-associated ophthalmopathy
GO: Graves’ ophthalmopathy
EOMs: Extraocular muscles
OF: Orbital fat
TSHr: Thyrotropin receptor
TRAbs: Thyrotropin receptor autoantibodies
IGF-1: Insulin-like growth factor-1
IGF-1r: Insulin-like growth factor-1 receptor
EUGOGO: European Group of Graves’ Orbitopathy
CAS: Clinical Activity Score
DON: Dysthyroid optic neuropathy
CT: Computed tomography,
MeSH: Medical Subject Heading
SD: Standard deviation
IQR: Interquartile range
NOS: Newcastle–Ottawa Scale
CI: Confidence interval
HCs: Healthy controls
GC: Glucocorticoid
pSS: Primary Sjogren’s syndrome
IgG4-RD: IgG4-related disease
IgG4-ROD: IgG4-related ophthalmic disease
IONE: Infraorbital nerve enlargement
