# Supplementary material for: Magnetic resonance imaging parameters on lacrimal gland in thyroid eye disease: a systematic review and meta-analysis
Source: BMC Ophthalmol. 2023 Aug 8;23:347. doi: 10.1186/s12886-023-03008-x (PMC10408192; doi:10.1186/s12886-023-03008-x)
Supplement: Supplementary file 2 — Additional file 2. [file 12886_2023_3008_MOESM2_ESM.docx]

**Additional file 2**

$$\left( \text{1} \right)\text{: Estimated Mean}=\frac{Lower Quartile+Median+Upper Quartile}{3}$$

**Formula 1.** Formula for mean estimation.

$$\left( \text{2} \right)\text{:Estimated SD}=\frac{Upper Quartile-Lower Quartile}{1.35}$$

**Formula 2.** Formula for SD estimation.

$$\text{Sample size = }N_{1}+N_{2}$$

$$\text{Mean = }\frac{N_{1}M_{1}+N_{2}M_{2}}{N_{1}+N_{2}}$$

$$\text{SD = }\sqrt{\frac{{(N}_{1}-1){SD}_{1}^{2}+(N_{2}-1){SD}_{2}^{2}+\frac{N_{1}N_{2}}{N_{1}+N_{2}}(M_{1}^{2}+M_{2}^{2}-2M_{1}M_{2})}{N_{1}+N_{2}-1}}$$

**Formula Set 3.** Formulae for combining sample size, mean and SD of two groups. *N­­­_1_*=sample size of group 1; *N­­­_2_*=sample size of group 2; *M­­­_1_*=mean of group 1; *M­­­_2_*=mean of group 2; *SD_1_*=standard deviation of group 1; *SD_2_*=standard deviation of group 2.
